# Malnutrition and Nutrition-Therapy: Our Neglected Responsibility

**DOI:** 10.1155/2011/842085

**Published:** 2011-07-18

**Authors:** Irit Chermesh, Lubos Sobotka, Corina Hartman, Rémy Meier

**Affiliations:** ^1^Technion-Israel Institute of Technology, Haifa 32000, Israel; ^2^Charles Universityin prague, prague, Czech Republic; ^3^Schnider Children's Medical Center of Israel Petack Tikuah 49202, Israel; ^4^Gastroenterology, Hepatology and Nutritional Department, University Clinic, Kantonsspital Liestal, 4410 Liestal, Switzerland

Around 30% of hospitalized patients in Europe are malnourished, and the figures for the community are also alarming. In certain diseases the proportion of malnourished patients can go up to 60%. The consequences of malnutrition are still neglected. The levels of knowledge and awareness of nutritional problems are low among all caregivers. Malnutrition is a heavy burden for the society, leading to increased morbidity, longer hospital stays, increased complications, decreased quality of life for the patients, and higher costs. Interventions to ensure appropriate nutritional care would be cost-effective. The impact of nutritional support is well known from many clinical trials. For each patient group specific nutritional recommendations are published in several reviews and guidelines. Although it is known how to do better, the nutritional support is often not regarded as an important therapeutic tool of the patients. 

In this issue several interesting papers summarize different important aspects related to malnutrition and nutritional support. 

The first paper describes the mechanism of cancer cachexia and the clinical implication and helps to understand why nutrition should be a central part in the management of cancer patients. It is well known that with an adequate nutritional support an increase of the quality of life can be achieved.

Two further papers are dealing with nutritional aspects in the elderly. A large survey in Germany confirms the high prevalence of malnutrition in nursing homes. A significant number of orally and tube-fed patients were malnourished. The most important factors leading to malnutrition were analysed. 

Oropharyngeal dysphagia is a high prevalent problem in the elderly and is a leading factor for malnutrition and aspiration. The paper in this issue gives important information for diagnosing and the treatment for this clinically relevant problem.

Three papers are related to surgical and ICU patients. Too often malnutrition is neglected in surgical patients. The clinical outcome is significantly different in malnourished patients compared to well-nourished controls. The paper on malnutrition in surgery wards confirms the importance of screening all surgical patients. 1/3 of the reported patients were malnourished, and the outcome was not as good compared to the patients not at nutritional risks. Elective surgery should be avoided until the nutritional deficits are corrected. In a further paper the perioperative nutritional support is described.

Enteral nutrition was not given for long time to patients with acute pancreatitis because of the fear of worsening the outcome. This opinion has changed in the last decade. The importance and limits of enteral nutrition are well explained in this issue.

In other three papers more general nutritional items are addressed. In the ICU hyperglycaemia is associated with poor outcome. Several interesting trials were published in the past on this specific problem. Until now the best blood sugar control is still debated. The review in this issue helps the reader to understand this controversial problem in more detail. 

 In patients with severe malnutrition it is important to know that nutrition support can also be harmful if the refeeding syndrome is not considered. The paper on the refeeding syndrome is therefore very helpful for understanding and avoiding the refeeding problems.

In nutritional practice the placing of a percutaneous endoscopy gastrostomy is very common. This procedure is done more and more on a propofol-based sedation. There are only few data on the safety of propofol-based sedation in the PEG procedure. The paper in this issue shows that propofol is a safe procedure if it is done according to the common guidelines. There is no significant difference in overall complication rates, sedation, and procedure-related complication.

Obesity is also regarded as a form of malnutrition. Controlled weight loss is well recognized as to be beneficial to reduce complications in these patients. One paper in this issue reports data on a specific supplement on the effect on weight loss. The use of green coffee extract shows some promising effects but the available trials are of poor methodological quality. A general recommendation cannot be given now.

The last paper is dealing with the important unhealthy Western diet. This diet is too high in n-6 polyunsaturated fatty acids (PUFA) and too low in n-3 PUFAs. We know that these diets can have negative effects on health. It is extremely important that all efforts should be undertaken to decrease this unhealthy diet in the future. Until now these important recommendations are not sufficiently implemented in the daily practice. This paper is therefore very important for promoting general health. 

We hope that the selected manuscripts help the readers to understand more the importance of recognizing the burden of malnutrition and to raise the awareness of the impact of nutritional support for the patients. 

*Irit Chermesh* *Irit Chermesh*

*Lubos Sobotka* *Lubos Sobotka*

*Corina Hartman* *Corina Hartman*

*Rémy Meier* *Rémy Meier*

